# Identification of Key Genes Regulated by Lactylation Modification and Associated with Tumor Immune Microenvironment in Breast Cancer

**DOI:** 10.3390/cimb48040416

**Published:** 2026-04-17

**Authors:** Yaohong Xie, Yi Ge, Na Miao, Pengxia Zhang, Jiaqi Xia

**Affiliations:** Key Laboratory of Microecology-Immune Regulatory Network and Related Diseases, School of Basic Medicine, Jiamusi University, Jiamusi 154007, China; xieyhjon@icloud.com (Y.X.); yige@stu.njmu.edu.cn (Y.G.)

**Keywords:** breast cancer, lactylation, single cell-seq, tumor immune microenvironment

## Abstract

Breast cancer (BRCA) is the most common cancer worldwide, with an incidence exceeding that of lung cancer. Protein lactylation, a newly identified post-translational modification involving the binding of lactic acid to lysine residues, plays an important role in BRCA. However, its role in BRCA progression remains largely unexplored. This study aims to identify and characterize the lactylation-related genes involved in BRCA biology. Transcriptomic and clinical data of BRCA and normal breast tissues were obtained from TCGA and GEO. Lactylation-related genes were curated from literature and intersected with BRCA datasets to identify candidates. A prognostic risk model was constructed using LASSO and Cox regression. Functional enrichment was performed using KEGG, GSVA, and GSEA. Immune correlations were evaluated by ESTIMATE, CIBERSORT. Single-cell RNA-seq data were integrated to assess gene expression heterogeneity across tumor and immune compartments. In vitro, MDA-MB-231 cells were treated with sodium L-lactate and lactylation-inducing agents, and gene expression was validated by Western blot and RT-qPCR, while EdU and wound healing assays evaluated proliferation and migration. We identified six hub genes associated with the immune microenvironment. Notably, S100A4 is significantly underexpressed, suggesting their potential regulatory roles in BRCA. Further analysis demonstrated that lactylation-related genes are closely linked to immune regulation in BRCA, indicating a possible crosstalk between metabolic modification and tumor immunity. Additionally, we found that lactylation significantly influences gene expression patterns and immune infiltration in BRCA. Importantly, lactic acid ions were shown to upregulate lactylation levels in BRCA cells, underscoring the functional impact of metabolic signals on post-translational modifications in tumorigenesis. Our findings indicate a potential mechanism wherein lactylation affects BRCA progression via lactic acid-driven regulation of the immune microenvironment; they also highlight the possible involvement of S100A4 in this process and offer new insights that could contribute to the diagnosis and treatment of BRCA.

## 1. Introduction

Breast cancer (BRCA) is a common cancer that affects women around the world [1]. Every minute, four women are diagnosed with BRCA worldwide and one woman dies from the disease [2], and these statistics are worsening. BRCA became the most common type of cancer in the world in 2025, with an incidence rate exceeding lung cancer. Despite the continuous advancement of diagnostic and treatment strategies, improving recurrence-free and overall survival remains a challenge [3]. Based on molecular and histological evidence, BRCA can be divided into three categories: BRCA that expresses hormone receptors (estrogen receptors (ER) or progesterone receptors (PR)), BRCA that expresses human epidermal receptor 2 (HER2), and triple-negative breast cancer (TNBC) [4,5]. The exact mechanism of BRCA initiation is not yet known [3,6]. It is reported that 20–30% of BRCA patients may experience metastasis after diagnosis and primary tumor treatment, and approximately 90% of cancer-related deaths are attributed to metastasis [7]. BRCA exhibits a metastatic tendency to different organs—including bone, lung, liver, and brain—called metastatic heterogeneity, and leads to diversified responses to treatment and patient prognosis [8,9]. Resistance to chemotherapy, radiotherapy and endocrine therapy is the main obstacle to maintaining patient survival. Furthermore, the intricate interactions between cancer-associated fibroblasts (CAFs) and immune cells within tumor microenvironment (TME) of BRCA significantly contribute to tumor progression and therapeutic resistance. However, the presence of immune tolerance mechanisms, such as the activation of myeloid-derived suppressor cells (MDSCs), along with treatment-related adverse effects, notably limit the efficacy of immunotherapy [10,11]. Therefore, the exploration of new immune-related markers is crucial to improving BRCA patient outcomes. This body of research indicates that immunotherapy plays a crucial role in improving survival outcomes for patients with BRCA.

Protein lactylation is a newly discovered post-translational modification of proteins involving the binding of Lactylation modification of acid molecules to lysine residues, first reported in 2019. This modification exists in a variety of human tissues and plays an important role in biological processes such as glycolysis, macrophage polarization, neurodevelopment and tumor proliferation. Accumulating evidence highlights the critical role of lactic acid metabolism in various types of cancer [12]. For example, DCBLD1 lactylation at lysine 172 suppresses G6PD autophagic degradation, activates the pentose phosphate pathway, and promotes cervical cancer progression [13]. H3K18 lactylation enhances the expression of the oncogene lipocalin-2, promoting bladder cancer [14]. Lactylation of nucleolin, particularly at lysine 477, regulates the RNA splicing of MAP kinase-activating death domain protein (MADD), prevents nonsense-mediated mRNA decay, and increases MADD expression in cholangiocarcinoma cells, thereby promoting intrahepatic cholangiocarcinoma progression [15]. In the triple-negative BRCA heterogeneity model, lactylation modification may be involved in TME remodeling and metabolic reprogramming processes, providing a new perspective for understanding tumor cell adaptability and drug resistance mechanisms, suggesting that it is a potential therapeutic target possibility [16]. Previous studies have revealed that PRDX1-positive monocytes play a significant role in BRCA through enhanced intercellular communication and immune regulatory functions, thereby influencing TME and facilitating the development of effective survival prediction models [17]. Additionally, research on the role of lysine lactylation (Kla) in BRCA has suggested that Kla may serve as a novel therapeutic target with significant impacts on prognosis, immunotherapy, and drug responses [18]. In summary, investigations into TME interactions and lactylation modifications in BRCA may offer new avenues for therapeutic advancements. Lactylation promotes the formation of an immunosuppressive microenvironment within tumors by enhancing M2 macrophage polarization and increasing the expression of immunosuppressive molecules such as IL-6, IL-10, and iNOS, thereby supporting tumor progression and metastasis [19]. Additionally, lactylation plays a role in regulating the function of tumor-associated immune cells by promoting the generation of Treg cells and inhibiting NK cell activity, which underscores its significance in tumor immune evasion and offers new perspectives for cancer immunotherapy based on metabolic reprogramming [20,21,22].

Emerging evidence highlights the prognostic significance of lactylation-related genes in both breast and gastric cancers. However, the specific functional roles and underlying mechanisms of protein lactylation in BRCA progression remain poorly understood [23,24]. Notably, the potential interplay between lactylation, the tumor immune microenvironment, and response to immunotherapy has not been thoroughly explored. Given the growing recognition of lactylation as a critical post-translational modification in cancer biology, further investigation into its physiological and pathological implications in BRCA is warranted. This study aims to elucidate the functional relevance of lactylation in BRCA progression and evaluate its potential impact on therapeutic strategies.

## 2. Materials and Methods

### 2.1. Data Acquisition and Data Preprocessing

We obtained the expression in BRCA and corresponding clinical data from TCGA (https://portal.gdc.cancer.gov/projects/TCGA-BRCA, accessed on 3 April 2025), which contains 113 healthy individual samples and 1118 BRCA patient samples. The gene mutation data of BRCA patients were also downloaded from the TCGA database. We downloaded three datasets from the GEO database, which are GSE42568, GSE118389, and GSE161529 (GEO database https://www.ncbi.nlm.nih.gov/geo/, accessed on 3 April 2025). A total of 332 lactylation-related genes were obtained from the relevant literature [25]. We also downloaded single-cell sequencing data for BRCA and normal samples from the GEO database. Following data acquisition, rigorous data cleaning and standardization procedures were performed. TCGA RNA-seq data were normalized to transcripts per million values and subsequently log2-transformed. GEO datasets were processed using log2 transformation according to their respective platform annotations. Since all datasets were analyzed independently rather than integrated, batch effect correction was not applied. First, samples containing outliers or missing data are removed and the remaining data are normalized to eliminate the impact of technical differences. Subsequently, we performed bioinformatics analysis to identify hub genes and their pathways. The overall difference in gene expression between BRCA patients and healthy individuals was compared using the “limma” package (limma_3.62.2); the screening criteria were set to |log2 (FoldChange)| > 1 and adjusted *p*-value < 0.05. The Benjamini–Hochberg (BH) method was applied to control the false discovery rate (FDR). Immune cell infiltration was estimated using the CIBERSORT algorithm, and only samples with *p*-value < 0.05 were retained for further analysis to ensure reliability. Correlation analysis was conducted using Spearman’s rank correlation coefficient, with *p* < 0.05 considered statistically significant.

### 2.2. Identify Hub Genes in BRCA Patients

We conducted an intersection analysis between the differentially expressed genes (DEGs) and those associated with lactylation. We utilized the GeneMANIA database (v12.0) to construct a protein–protein interaction (PPI) network based on these overlapping genes. The R packages “clusterProfiler” (clusterProfiler_4.12.0) and “org.Hs.eg.db” (org.Hs.eg.db_3.20.0) were utilized for conducting Gene Ontology (GO) enrichment analysis, which aimed to disclose the biological process (BP), cell component (CC), and molecular function (MF) enrichment of the DEGs. Subsequently, the enrichment analysis of Kyoto Encyclopedia of Genes and Genomes (KEGG) was carried out to explore the signaling pathways related to the 13 genes with differences in lactic acid. The significant pathway screening threshold for the KEGG signaling pathway is defined as *p* < 0.05. The hub gene was selected by LASSO (minimum absolute contraction and selection operator) regression for feature selection and achieved by R package “glmNETs” (glmNETs_4.1-8). When building a regularization model, set alpha = 1 and use 10-fold cross-validation to determine the optimal λ value (λ.min standard). The non-redundant gene set with the strongest predictive power for the target phenotype was selected using the penalty function of the contraction coefficient to zero [26].

### 2.3. Relationship Between Lactylation Hub Genes and Immune Characteristics

Gene set variation analysis (GSVA) calculated the variations enriched by gene sets in the TCGA dataset in an unsupervised manner and scored the gene sets of individual samples. We used the R package “GSVA” (GSVA_1.54.0) to analyze the correlation between the expression of the hub gene and the KEGG signaling pathway in the samples. Meanwhile, the ssGSEA algorithm was also used to comprehensively evaluate the differences in immune cell infiltration of each sample. Using Spearman correlation analysis, the association between core genes and infiltrating immune cells was explored.

### 2.4. Evaluation of Tumor Microenvironment and Immune Cell Composition

ESTIMATE is a method for determining the fractions of stromal cells and immune cells based on the gene expression characteristics in tumor samples. We evaluated tumor immune microenvironment of each BRCA patient by using this algorithm. CIBERSORT is a method for calculating the composition of cells based on expression profiles. This deconvolution algorithm was used to calculate the proportion of 22 immune cells in each BRCA patient. Finally, a correlation heat map of immune cell infiltration was made by using the R package “ggcorrplot” (ggcorrplot_0.1.4.1) to reveal the correlations among immune cells.

### 2.5. scRNA-Seq Analysis

The original data of 10X Genomics single-cell sequencing (scRNA-seq) was quality-controlled and standardized by using the R package “Seurat” (Seurat_5.1.0), and abnormal cells with mitochondrial gene proportion > 20% or with a detection gene number < 200 were eliminated. Highly variant genes (Top 3000) were selected for downstream analysis. Principal component analysis (PCA) was used for dimensionality reduction, cell clustering was used (resolution = 0.6) to cluster cells, and cell subpopulations were identified. Finally, DEGs were identified by using the FindAllMarkers function (Wilcoxon test, min.pct = 0.25), and cell types were determined by referring to the CellMarker2.0 database [27].

### 2.6. Cell Culture

Human BRCA cells (MDA-MB-231, Wuhan Punosai Biotechnology Company, Wuhan, China) were cultured in Dulbecco’s Modified Eagle Medium (DMEM) supplemented with 10% fetal bovine serum (FBS), 1% penicillin–streptomycin, and maintained at 37 °C in a humidified atmosphere containing 5% CO_2_. The medium was changed every two days, and the cells were subcultured when the cell growth density reached 80%. At passage 4–5, we added different concentrations of exogenous lactylation modification acid and sodium L-lactate into the cell culture at different times and then extracted protein or RNA. In addition, we guarantee that when the cells are stable to 7–8 passages, we will perform drug treatments [28].

### 2.7. Drug Treatment

MDA-MB-231 cells were treated with lactic acid or sodium L-lactate at concentrations of 0 mM, 12.5 mM, 25 mM, and 50 mM under two experimental conditions: different drug concentrations for a fixed incubation time of 12 h, and a fixed drug concentration of 25 mM for varying incubation times (0 h, 3 h, 6 h, and 12 h). All treatments were performed in triplicate to ensure reproducibility [29]. Lactic acid and sodium L-lactate were purchased from Sigma Company (St. Louis, MO, USA), with the CAS Number of lactic acid: 79-33-4, and the CAS Number of sodium L-lactate: 867-56-1.

### 2.8. Protein Extraction and Western Blot

Cells were lysed in RIPA buffer containing protease and phosphatase inhibitors. Protein concentration was quantified using a BCA Protein Assay Kit (Thermo Fisher Scientific, Waltham, MA, USA). Equal amounts of protein (30 µg) were separated by SDS-PAGE and transferred onto PVDF membranes. Membranes were blocked with 5% non-fat milk in TBST for 1 h at room temperature and then incubated overnight at 4 °C with primary antibodies against target proteins (Beta Tubulin Recombinant antibody, 1:10,000; Anti-L-Lactyl Lysine Rabbit mAb, 1:1000; S100A4 Polyclonal antibody, 1:5000). After washing, membranes were incubated with horseradish peroxidase-conjugated secondary antibodies for 1 h at room temperature. Protein bands were visualized using an enhanced chemiluminescence (ECL) detection system, and densitometric analysis was performed using ImageJ (ImageJ 1.54f) software.

### 2.9. Wound Healing Assay

We use a ruler to draw three parallel lines on the back of the 6-well plate, seed the cells into the 6-well plate and grow to confluence, then use a sterile pipette 200 μL tip to scratch in a straight line on the vertical back. Wash the cells twice with PBS to remove the detached cells and treat with lactylation modification acid or sodium L-lactate as described above. Images of the scratched area were captured using an inverted microscope (Olympus IX71, Olympus, Tokyo, Japan)) at 0 h, 12 h, and 24 h.

### 2.10. RNA Extraction and RT-qPCR Analysis

Total RNA was extracted using TRIZOL reagent (Thermo Fisher Scientific) according to the manufacturer’s instructions. RNA concentration and purity were determined using a NanoDrop spectrophotometer (Thermo Fisher Scientific, Waltham, MA, USA). Complementary DNA (cDNA) was synthesized from 1 µg of total RNA using a reverse transcription kit (PrimeScript RT Reagent Kit, Takara, Shiga, Japan). Quantitative real-time PCR (RT-qPCR) was performed on a CFX96 Real-Time PCR Detection System (Bio-Rad, Hercules, CA, USA) using SYBR Green Master Mix. The relative expression levels of target genes were normalized to the housekeeping gene GAPDH and calculated using the 2^−ΔΔCt^ method. All treatments were performed in triplicate to ensure reproducibility. The sequences of the primers used in this study are provided in Table 1. The PCR primers are shown below (Table 1).

### 2.11. EdU Incorporation Assay

Cell proliferation was assessed using an EdU incorporation assay kit (EdU Cell Proliferation Kit, Beyotime Biotechnology, Shanghai, China). Briefly, cells were seeded into 12-well plates and treated with drugs. EdU labeling solution was added to the culture medium for 2 h. After fixation and permeabilization, cells were stained with Apollo^®^ dye (Guangzhou, China) for EdU detection and Hoechst 33342 for nuclear staining. Fluorescent images were acquired using a fluorescence microscope (Leica Microsystems, Wetzlar, Germany)..

### 2.12. Statistical Results Graphing and Statistical Analysis

Graphpadprism and ImageJ software were used for experimental data analysis and drawing. The *t* test was used for comparison between two groups, and the one-way ANOVA test was used for comparison between multiple groups. *p* < 0.05 was considered statistically significant. Each experiment was repeated three times, and it was statistically significant.

## 3. Results

### 3.1. Overall Expression Profile of Patients with Breast Cancer

**Figure 1:**
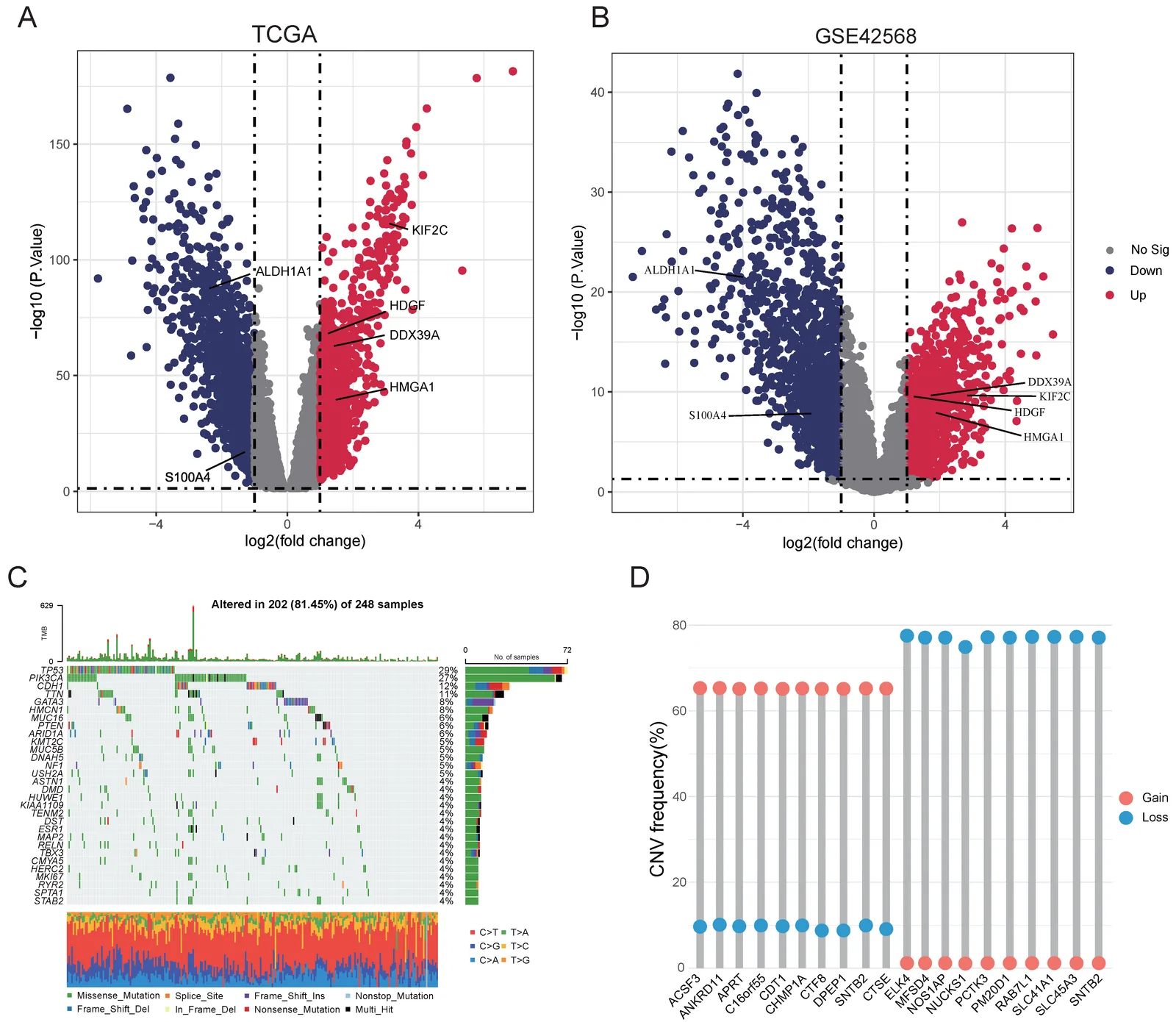
Comprehensive analysis of differential genes and genetic variants in breast cancer. (**A**,**B**) Analysis of DEGs in both TCGA and GEO datasets. The vertical dashed lines represent the log2 fold change cutoffs of ±1 (indicating a 2-fold change), and the horizontal dashed line represents the significance threshold of *p* < 0.05 (or FDR < 0.05). (**C**) Analysis of the top 20 high-frequency genes for point mutations (SNPs). (**D**) Frequency of the top 20 high-frequency genes for CNVs.

For our comparative analysis of BRCA versus non-cancer tissues across the TCGA and GSE42568 dataset, we identified a number of DEGs that may play a key role in the occurrence and development of BRCA in TCGA. There were 1397 downregulated genes and 914 upregulated genes, while in the GEO database, there were 1428 downregulated genes and 1336 upregulated genes (Figure 1A,B). Among these, we identified several lactylation-related genes, including *ALDH1A1, KIF2C, HDGF, DDX39A, HMGA1, S100A4, NUCKS1*, etc. Additionally, we performed clustering analysis using the TCGA database and GSE42568 to further characterize all gene expression profiles (Figure S1A,B). We also analyzed gene mutations and CNVs at the genetic level. The results of the gene mutations show the top 30 genes with the highest gene mutation frequency for BRCA (Figure 1C). At the same time, we analyzed the top 20 gain and loss genes for CNVs. We found that CNVs were more prevalent than point mutations in BRCA (Figure 1D). Among these genes, *NUCKS1*, primarily the “loss” gene, associated with lactylation, was located on chromosome 4. Therefore, we hypothesize that there is also a correlation between lactylation and CNVs. Therefore, we performed GO and KEGG analyses using differentially differentiated genes from two databases. GO analysis of the TCGA database showed that lactylation-related pathways, including cell cycle regulation, acting cytoskeletal organization, and extracellular matrix organization, were significantly enriched in BRCA development (Figure S1C,E). The GO analysis identified that the lactylation-associated pathways such as organic acid catabolic processes, fatty acid metabolic processes, and extracellular matrix organization were significantly enriched in the GEO dataset (Figure S1D,F). Therefore, we conducted this study on lactylation and its role in BRCA.

### 3.2. Expression Characteristics of Lactylation-Related Genes

**Figure 2:**
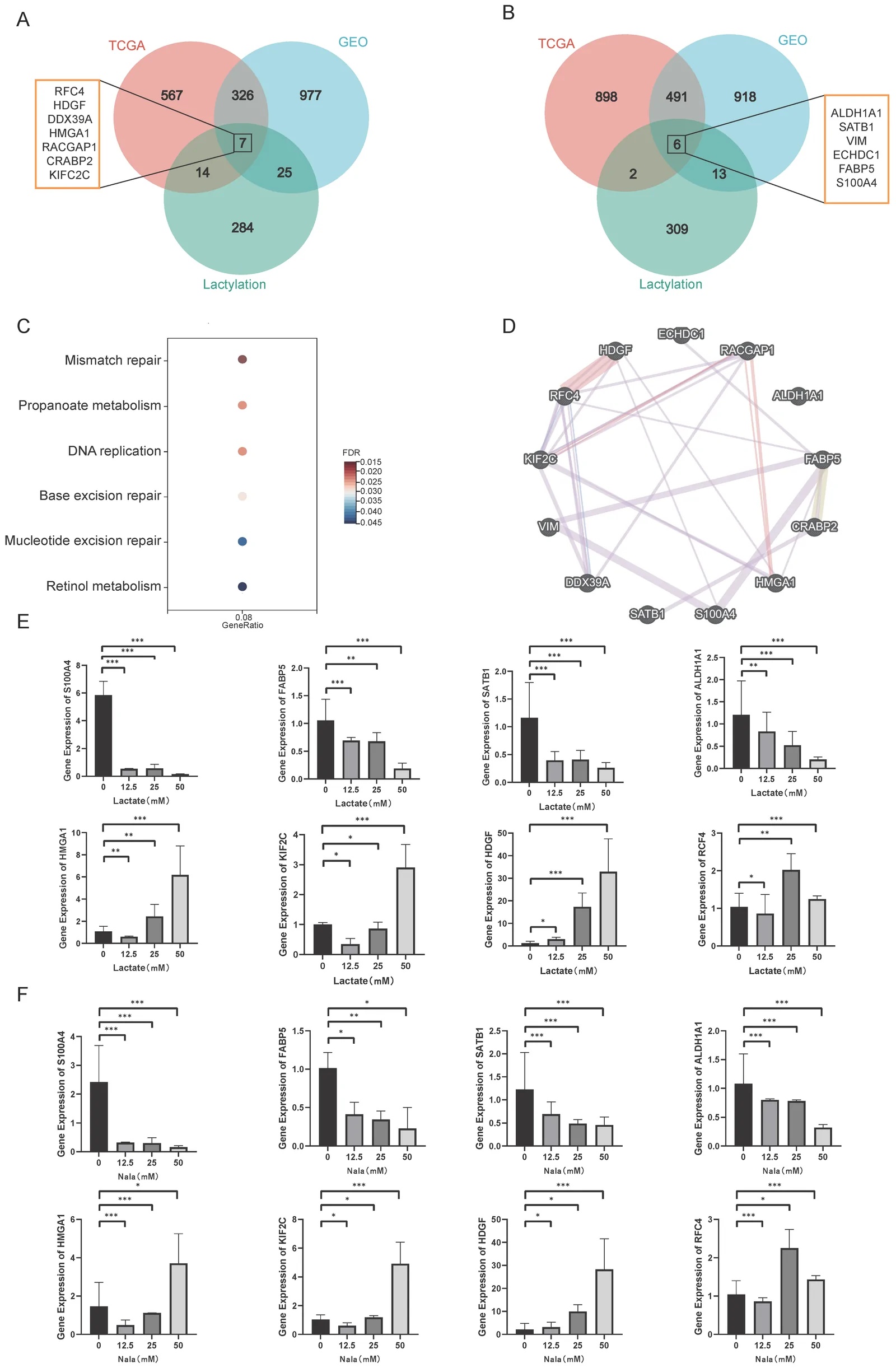
The expression characteristics of lactylation-related genes. Venn diagram shows the intersection of (**A**) upregulated genes and (**B**) downregulated genes with lactylation genes. (**C**) KEGG analysis of 13 differentially expressed lactylation-related genes. (**D**) Interaction network of proteins using the GeneMANIA database. (**E**) Validated RNA levels of lactic acid-treated cells using RT-qPCR technology. (**F**) Validated RNA levels of sodium L-lactate -treated cells using RT-qPCR technology. *, *p* < 0.05; **, *p* < 0.01; ***, *p* < 0.001.

To investigate the expression characteristics of lactylation-related genes, we identified and collected 332 lactylation genes. As illustrated in Figure 2A, the intersection of these upregulated DEGs across the three datasets revealed seven overlapping and consistently upregulated genes, *RFC4, HDGF, DDX39A, HMGA1, RACGAP1, CRABP2 *and* KIF2C*, while six lactylation-related genes were downregulated in BRCA patients (*ALDH1A1, SATB1, VIM, ECHDC1, FABP5, S100A4*) (Figure 2B). Notably, although S100A4 was identified as downregulated in the bulk RNA-seq analysis, this observation may reflect the average expression across heterogeneous tumor tissues. Given the cellular heterogeneity of the TME, S100A4 may exhibit elevated expression in specific cell populations, such as stromal or cancer-associated fibroblast compartments. Functional enrichment analysis revealed that these genes were significantly enriched in multiple biological processes and signaling pathways that may be closely associated with the lactylation modification pattern, including “Transcription repressor complex”, “Nuclear matrix”, “ldehyde dehydrogenase (NAD+) activity”, “Organic acid transport”, etc. (Figure S2A). Functional enrichment analysis revealed that these genes were significantly enriched in biological processes such as mismatch repair, propanoate metabolism, and DNA replication, which may be closely related to immune responses and metabolic regulation in TME. This suggests that lactylation and immunity are closely related. This provides an important research direction for the relationship between lactylation modification. To further explore the correlation of 13 genes, we constructed the protein interaction network and observed a strong interconnection among them (Figure 2D).

We performed RT-qPCR to validate the expression levels of these 13 candidate genes in MDA-MB-231 cells. The results demonstrate that eight genes (*S100A4, FABP5, SATB1, ALDH1A1, HMGA1, KIF2C, HDGF, RFC4*) exhibit significant differences in expression across experimental groups with varying drug concentrations or treatment durations (Figure 2E,F). Notably, the expression levels of these genes under 0 μM drug treatment were aligned with those observed in our bioinformatics analysis. Among the core genes, S100A4 displayed the most remarkable expression change and has been well-documented to play a critical role in cancer progression. Therefore, S100A4 will be prioritized as a key target for subsequent functional studies. Among the core genes, S100A4 showed the most significant expression change and is known to be involved in cancer progression. Therefore, S100A4 will be a key target for further study. In contrast, the remaining five genes (*CRABP2, DDX39A, RACGAP1, VIM, ECHDC1*) showed less pronounced changes than anticipated (Figure S2B,C).

### 3.3. Lactylation-Related Hub Genes in BRCA Patients

**Figure 3:**
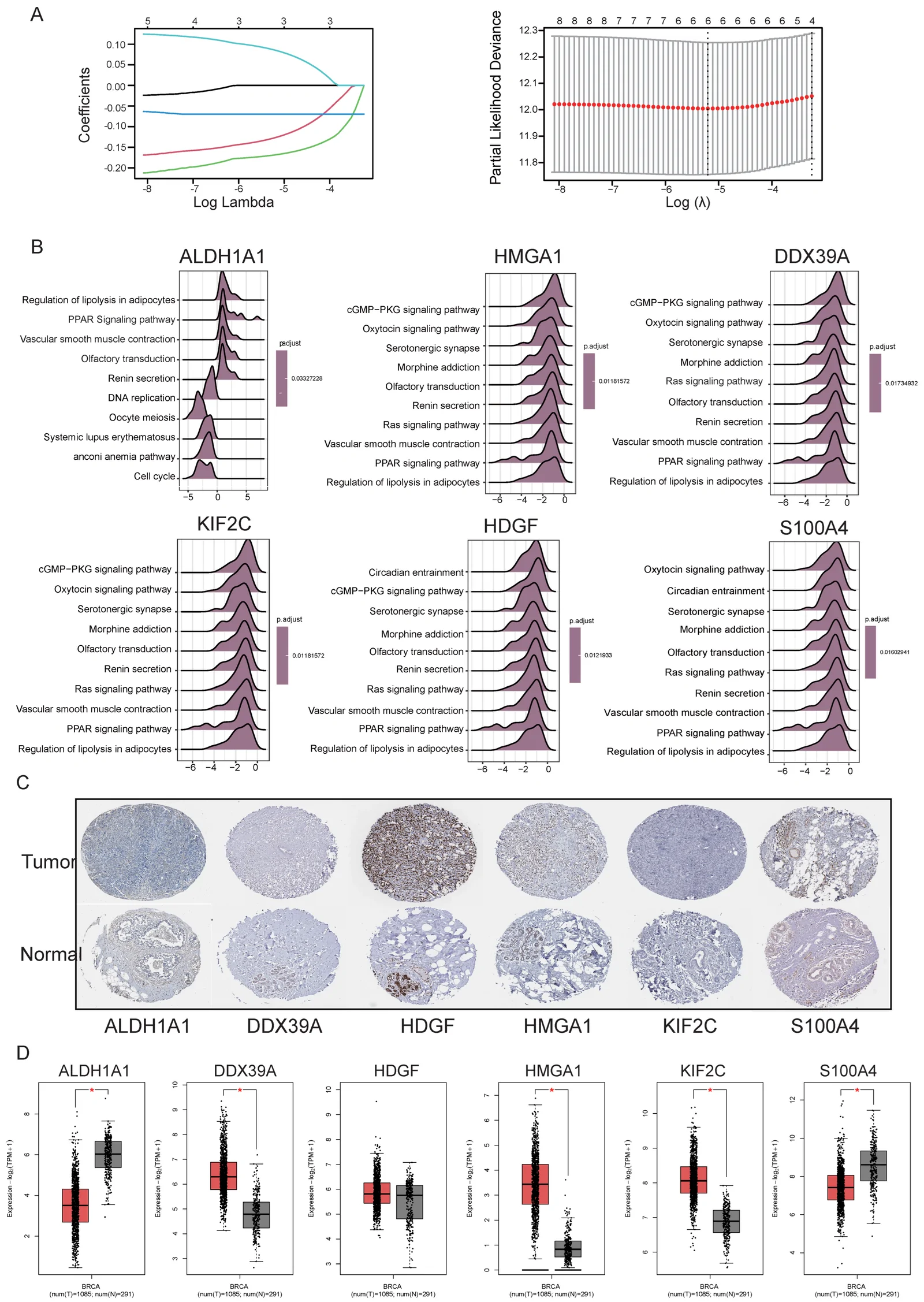
Screening for hub lactylation-related genes and analyzing their immune pathway in breast cancer. (**A**) LASSO coefficient profiles of candidate genes. Each colored line represents the coefficient trajectory of an individual gene as the penalty parameter (log λ) varies. The six curves correspond to the six selected hub genes (*ALDH1A1, HMGA1, DDX39A, KIF2C, HDGF, *and* S100A4*). (**B**) The GSEA analysis of KEGG (top 10) based on the correlation analysis result of ALDH1A1, HMGA1, DDX39A, KIF2C, HDGF and S100A4, Scale bar: 100 μm. (**C**) Immunohistochemistry of the six hub genes in BRCA pathological tissues. (**D**) Expression levels of the six lactylation-related genes in cancer and normal samples. *, *p* < 0.05.

The lactylation-related signature composed of six hub genes (*ALDH1A1, DDX39A, HDGF, HMGA1, KIF2C, *and* S100A4*) were established using 1000 iterations of LASSO regression analysis (Figure 3A). Given the focus on the relationship between lactylation and immune functions, GSEA analyses indicate that the expression levels of genes such as *ALDH1A1, DDX39A, HDGF, HMGA1, *and* KIF2C* are significantly correlated with the infiltration of various immune cell types, particularly playing important roles in the regulation of the tumor immune microenvironment (Figure 3B). Notably, several of these genes exhibit a negative correlation with CD8^+^ T cells, suggesting that they may facilitate tumor immune escape by promoting the infiltration of immunosuppressive cells or suppressing the activity of effector T cells, thus providing potential therapeutic targets for immunotherapy strategies in breast cancer. As illustrated in Supplementary Figure S3A, there are significant differences in the expression levels of these genes between the high- and low-immune infiltration groups, suggesting their potential involvement in regulating the tumor immune microenvironment. These findings indicate that the genes may possess immunologically relevant biological functions and hold clinical research value. At the same time, prognostic survival analysis of six hub genes in BRCA indicates that the six individual genes have no effect on survival and that it is the combined action of all six genes that contributes to the survival outcome (Figure S3B). Figure 3C shows that the expression levels of HDGF, HMGA1, and S100A4 are significantly upregulated in BRCA tissues compared to normal tissues, based on data from the HPA database, confirming that the screened genes play important roles in BRCA progression and may serve as potential therapeutic targets or directions for drug development. Bioinformatics analysis revealed that the expression levels of ALDH1A1, DDX39A, HMGA1, KIF2C, and S100A4 were significantly elevated in BRCA tissues compared to normal tissues (Figure 3D). The immunohistochemistry results of the hub genes obtained from the HPA database were consistent with the bioinformatics analysis findings. Therefore, we further investigated the role of these hub genes in the tumor immune microenvironment in breast cancer.

### 3.4. Lactylation Affects the Tumor Immune Microenvironment in BRCA

**Figure 4:**
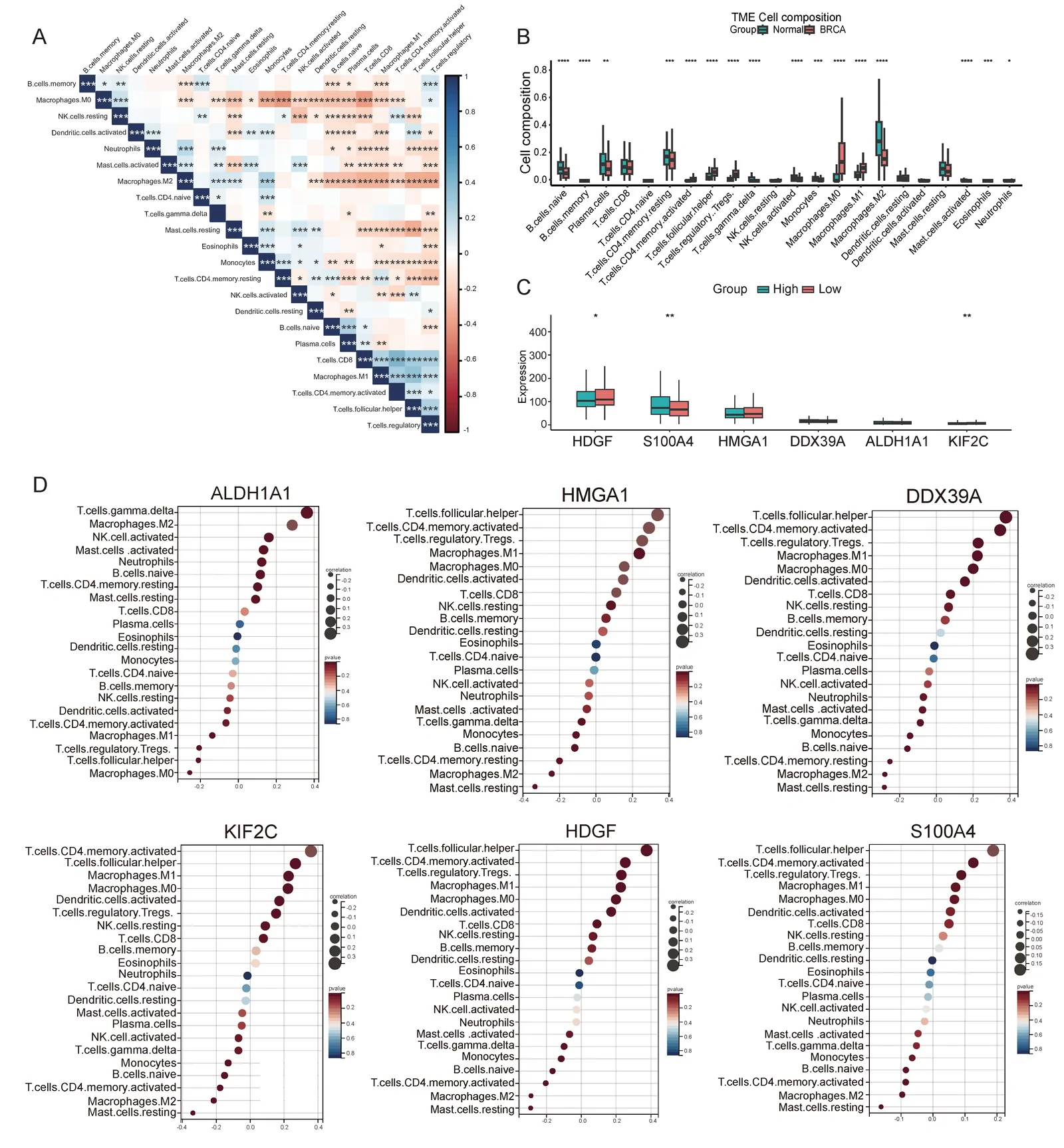
Correlation of hub genes with immune cell infiltration. (**A**) The correlation of the abundance of 22 infiltrating immune cell types. *, *p* < 0.05; **, *p* < 0.01; ***, *p* < 0.001; ****, *p* < 0.0001. (**B**) The proportion of immune cells between BRCA and normal samples. (**C**) Lactylation-related gene expression in the two risk groups. (**D**) Demonstration of correlation between immune cell infiltration and ALDH1A1, HMGA1, DDX39A, KIF2C, HDGF and S100A4.

To further clarify the role of lactylation-related hub genes in the TME of BRCA, we used the Cibersort algorithm to obtain the infiltration of immune cells in each sample. As shown in Figure 4A, there were significant differences in the patterns of immune cell infiltration between BRCA patients and control samples. For example, we observed significant negative correlations between M0 macrophages and monocytes, CD4^+^ T cells, and B cells, as well as between M2 macrophages and plasma cells, CD4^+^ T cells, and regulatory T cells, TAM. Our analysis revealed that regulatory T cell infiltration was significantly elevated, while NK cell infiltration was markedly reduced in BRCA samples. Additionally, we observed increased immunosuppressive features and decreased monocyte signature scores in BRCA patients (Figure 4B). To analyze the association between the expression levels of six lactylation-related genes and immune infiltration, the immune infiltration level in the high-expression group of S100A4 was correspondingly higher, whereas HDGF showed the opposite trend (Figure 4C). Next, we analyzed the relationship between these six genes and immune-related pathways, as shown in Figure 4D, including S100A4 showed the strongest associations with the infiltration profiles of 14 immune cell subsets, particularly tumor-associated macrophages and MDSCs. However, S100A4 also displayed inverse correlations with regulatory T cells, suggesting its potential involvement in suppressing anti-tumor immune responses. HDGF shows a positive correlation trend with follicular helper T cells and a negative correlation with M2 macrophages. This indicates that the expression of HDGF is related to promoting the immune response (Figure 4D). This finding complements our previous analysis of immune cell interactions within the BRCA TME, suggesting that these hub genes may play a crucial role in modulating the immune microenvironment. By targeting these genes, we could potentially influence the regulatory or antagonistic interactions observed among immune cells, thereby offering promising therapeutic strategies for BRCA treatment.

### 3.5. Single-Cell Profiling of Immune Cell Composition in BRCA

**Figure 5:**
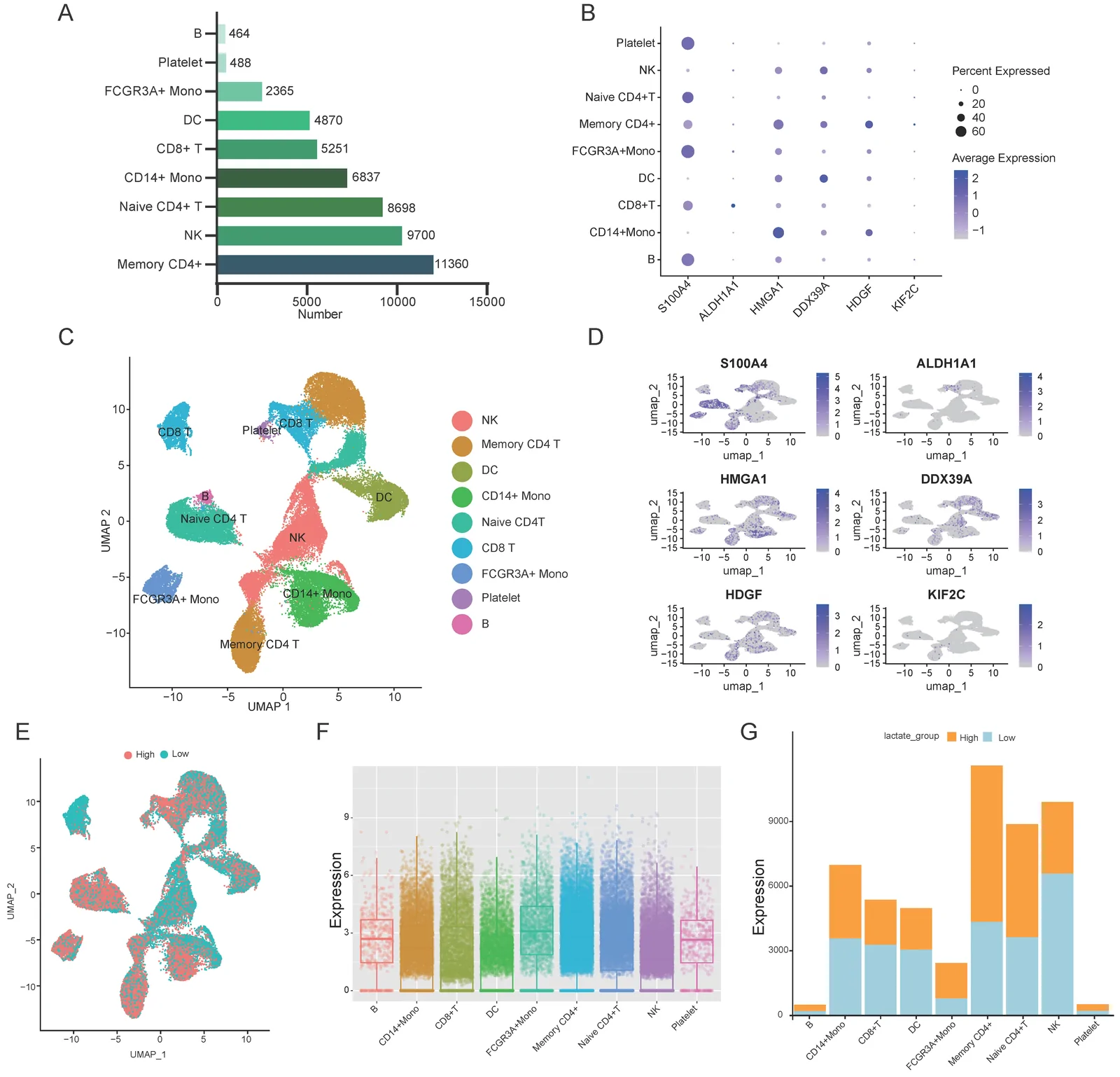
Single-cell sequencing analysis of BRCA. (**A**) UMAP plot showing immune cell clustering in BRCA and normal samples from GSE118389 and GSE161529 datasets, annotated into major immune cell types. (**B**) Dot plot showing the expression levels and proportion of cells expressing the core lactylation-related hub genes (*S100A4, ALDH1A1, HMGA1, DDX39A, HDGF, *and* KIF2C*) across annotated immune cell types. (**C**) UMAP-based re-clustering analysis of CD4^+^ T cells identify three distinct subclusters, indicating heterogeneity within this compartment. (**D**) Comparison of average lactylation signature scores among CD4^+^ T cell subclusters, highlighting differences in lactylation levels. (**E**) UMAP plots visualizing cell-type-specific expression of 6 lactylation-related genes, with high expression enriched in memory CD4^+^ T cells and NK cells. (**F**) Boxplots showing the summed expression of lactylation-related genes across different immune cell types at the single-cell level. (**G**) Distribution of high-lactic acid and low-lactic acid immune subpopulations based on composite lactylation scores.

To further elucidate the roles of lactylation-related hub genes in immune regulation within BRCA, we conducted single-cell RNA sequencing analysis using BRCA and normal breast tissue samples from the GSE118389 and GSE161529 datasets. After rigorous quality control and batch effect correction, many high-quality immune cells were retained for downstream analyses (Figure S4A,B). These cells were subsequently annotated into major immune cell types based on canonical marker expression and SingleR-based reference classification, including CD14^+^ monocytes, CD8^+^ T cells, naive and memory CD4^+^ T cells, dendritic cells, FCGR3A^+^ monocytes, NK cells, and platelets (Figure 5A,C). In contrast, only memory CD4^+^ T cells, CD14^+^ monocytes, and naïve CD4^+^ T cells were detected in normal tissues (Figure S4C). To explore the immune cell-specific expression of lactylation-related hub genes, we analyzed their distribution across immune cell types. Among BRCA-infiltrating immune cells, S100A4 exhibited significantly higher expression, whereas KIF2C and ALDH1A1 showed relatively low levels (Figure 5B,D). In normal tissues, the expression of hub genes remained relatively consistent across immune cell subsets (Figure S4D,E). These differences suggest that aberrant expressions of key lactylation-related genes—particularly S100A4 and HMGA1—may be associated with immune cell alterations in the BRCA microenvironment. To further investigate lactylation heterogeneity, we computed a total lactylation score by summing the expression of all lactylation-related genes in each single cell. As shown in Figure 5F, distinct immune cell subsets displayed varying lactylation levels. Notably, CD4^+^ T cells in BRCA tissues showed higher overall lactylation scores, while CD14^+^ monocytes in normal tissues exhibited relatively elevated lactylation levels. These findings indicate that lactylation is not uniformly distributed, but rather is enriched in specific immune cell populations depending on the tissue context.

We next stratified all immune cells into high- and low-lactylation groups using the median total lactylation score as a cutoff. The composition of immune cells differed markedly between these groups (Figure 5G). In BRCA tissues, the high-lactylation group was predominantly composed of CD4^+^ T cells, whereas in normal tissues, high-lactylation cells were enriched in CD14^+^ monocytes. These results suggest that altered lactylation patterns may contribute to reshaping the immune microenvironment in BRCA and could represent a regulatory mechanism linking metabolic reprogramming to immune cell function during tumor progression.

### 3.6. Lactylation Promotes BRCA Cell Proliferation

**Figure 6:**
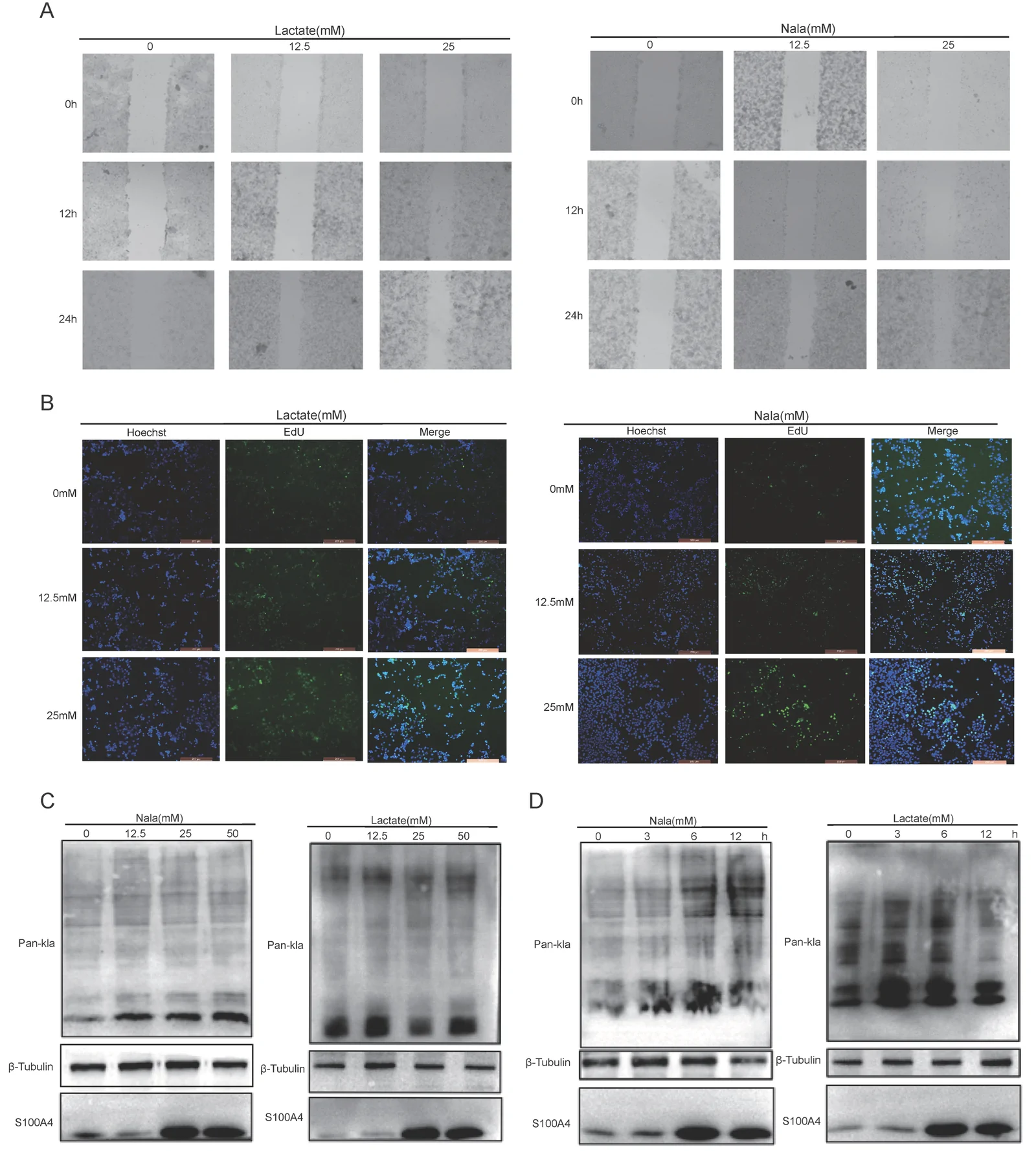
Lactic acid anion upregulates lactylation modification levels in breast cancer cells. (**A**) MDA-MB-231 cells were treated with 12.5 mM and 25 mM lactic acid/sodium L-lactate. Wound healing assay was used to assess cell migration. Scale bar: 200 μm. (**B**) MDA-MB-231 cells were treated with 12.5 mM and 25 mM lactic acid/sodium L-lactate. EdU staining was used to detect the proportion of positive cells, analyzing the effect on cell proliferation. Scale bar: 200 μm. (**C**) MDA-MB-231 cells were treated with various concentrations of lactic acid/sodium L-lactate (0, 12.5, 25, 50 mM) for 12 h, followed by Western blot to detect pan-Kla levels. (**D**) MDA-MB-231 cells were treated with 25 mM lactic acid and sodium L-lactate, and pan-Kla levels were detected by Western blot at 0 h, 3 h, 6 h, and 12 h.

Considering the critical involvement of lactylation in BRCA, we further explore how lactylation-mediated hub genes influence the progression of BRCA. In vitro experimental assays were performed to evaluate the functional roles of these key regulators in modulating cancer cell behavior through lactylation. We first treated MDA-MB-231 cells with exogenous lactic acid at various concentrations and conducted wound healing assays to observe the migration ability of BRCA cells under different lactylation levels. During the experiments, we noticed that the acidification effect caused by hydrogen ions dissociated from lactic acid molecules might influence the biological characteristics of BRCA cells. Therefore, we utilized sodium L-lactate as an exogenous treatment for BRCA cells to investigate changes in their biological properties while controlling pH effects. The results demonstrate that, with an increase in the concentration of exogenous lactic acid, the migration capability of BRCA cells gradually increased—a finding that was consistently observed in the sodium L-lactate experimental group as well. This suggests that lactic acid levels can directly impact the migratory behavior of BRCA cells, independent of pH changes (Figure 6A). The statistical test diagram of Figure 6 has been shown in Figure S5. Additionally, consistent with the effects observed using exogenous lactic acid, the proportion of proliferating BRCA cells gradually increased as the concentration of sodium L-lactate was incrementally raised. Therefore, by comprehensively analyzing the impact of both lactic acid and sodium L-lactate on BRCA cells, we have substantiated that the lactic acid anion can indeed modulate the biological characteristics of these cells (Figure 6B).

We subsequently detected changes in pan-KLA levels in BRCA cells by Western blot. The results show that a certain level of pan-Kla signal can be detected in BRCA cells without exogenous lactylation modification acid; with the increase in exogenous lactylation modification acid concentration, the level of pan-Kla in BRCA cells also tends to rise, indicating that the degree of lactylation modification acid modification rises (Figure 6C). Furthermore, we treated BRCA cells with the same concentration of exogenous lactylation modification acid and detected pan-KLA levels in cell samples at different time points. The results show that, as the treatment time of exogenous lactylation modification acid increases, the level of pan-Kla in BRCA cells also shows a trend increase (Figure 6D).

## 4. Discussion

BRCA remains the most prevalent malignant tumor among women worldwide, posing a significant threat to public health due to its high incidence and heterogeneity. Despite advancements in diagnostic techniques and therapeutic strategies, challenges such as therapy resistance, recurrence, and metastasis continue to impede effective disease control. In recent years, increasing attention has been directed toward tumor metabolism, particularly the role of lactic acid, a key metabolic byproduct of aerobic glycolysis [30], which is often upregulated in the TME. Accumulating evidence suggests that lactic acid is not merely a metabolic waste product, but functions as an immunomodulatory and signaling molecule that promotes tumor progression, immune evasion, and therapeutic resistance. Notably, the newly identified post-translational modification, histone lactylation, has emerged as a critical epigenetic mechanism linking cellular metabolism to gene regulation [31]. However, the role of lactylation modification in BRCA remains largely unexplored. Therefore, our study aimed to systematically investigate the expression characteristics, immune relevance, and functional implications of lactylation-related genes in breast cancer to elucidate their potential as prognostic biomarkers and therapeutic targets.

In this study, we comprehensively explored the landscape of lactylation in BRCA using multi-omics approaches, including bulk and single-cell transcriptomic analyses. We identified DEGs associated with lactylation by integrating TCGA and GEO datasets. Functional enrichment analyses revealed their involvement in cell proliferation, immune regulation, and metabolic reprogramming, suggesting that lactylation plays an essential role in BRCA progression. Using LASSO regression, we identified six hub genes: *ALDH1A1, HMGA1, DDX39A, KIF2C, HDGF, *and* S100A4*. We successfully constructed a prognostic model for breast cancer using these six genes, and, through in-depth analysis, we found that they have strong associations with BRCA prognosis, cancer-related signaling pathways, and immune infiltration. Several lactylation-related hub genes identified in our study have been previously reported to be associated with immune modulation in breast cancer, supporting the reliability of our results. For example, ALDH1A1 promotes MDSC expansion and showed a strong correlation with γδ T cells, suggesting that its lactylation might regulate immune recruitment. HMGA1 and DDX39A were both linked to T follicular helper (Tfh) cells, indicating a role in adaptive immunity regulation [32]. Among them, HMGA1 may participate in epigenetic regulation via lactylation, while DDX39A influences the immune contexture to promote BRCA progression. Conversely, genes such as HDGF and S100A4 were implicated in immunosuppression. HDGF suppresses CD4^+^ T cells and alters the TME [33], whereas S100A4, known for its role in metastasis and drug resistance, is highly expressed under lactic acid stimulation and potentially regulated through lactylation [34]. KIF2C, primarily associated with mitosis, has also been linked to migration, invasion, and chemoresistance, possibly through post-translational modifications [34].

Wound healing and EdU assays demonstrated that elevated lactic acid enhances BRCA cell proliferation by increasing lactylation levels. Western blot further confirmed that S100A4 protein is upregulated under high-lactic acid conditions, even when mRNA expression is low. This suggests lactylation may enhance S100A4 protein stability via post-translational mechanisms.

Previous research has shown that high lactylation scores are linked to a “cold” TME, characterized by reduced infiltration of CD8^+^ T cells, γδT cells, NKT, and NK cells. Single-cell RNA sequencing further revealed the heterogeneous expression of these hub genes across different immune and epithelial cell types within the TME in BRCA. Our study confirmed that high expression of these genes is associated with altered immune cell composition, especially in monocytes and T cell subsets. Our study expands on this by demonstrating that lactylation directly regulates gene expression and reshapes immune cell landscapes at the single-cell level. These findings highlight lactylation as a key regulatory node connecting metabolic rewiring and immune escape in BRCA.

While our integrative analysis provides valuable insights, several aspects merit further exploration. First, gene identification was primarily based on publicly available datasets and the literature, which may have limited the discovery of novel or less-studied candidates. Although the expression patterns of key genes were validated in vitro, additional in vivo experiments using animal models or patient-derived tissues would further strengthen our findings. Interestingly, we observed that MCF-7 cells exhibited rapid cell death upon lactic acid treatment, suggesting subtype-specific responses to lactic acid. This may be due to metabolic differences between luminal and TNBC subtypes, with TNBC cells showing higher glycolytic activity and lactic acid tolerance. Moreover, given that single-cell RNA sequencing reflects transcriptomic rather than proteomic states, it cannot directly capture lactylation modifications at the protein level. The apparent discrepancy in S100A4 expression between transcriptomic and experimental data may be attributed to tumor heterogeneity and multi-level regulation, including spatial expression differences and post-transcriptional modulation. Complementary approaches such as proteomics or ChIP-seq will be essential to elucidate the precise molecular mechanisms. Lastly, the potential therapeutic relevance of these hub genes should be further evaluated in well-designed preclinical studies.

Our study goes beyond descriptive bioinformatics analysis by proposing a potential mechanistic framework linking lactylation to tumor progression. Specifically, lactic acid accumulation in the TME may induce lactylation modifications, which in turn regulate key genes such as HMGA1 and S100A4, ultimately contributing to immune microenvironment remodeling in BRCA. The apparent inconsistency in S100A4 expression across different analyses may be attributed to tumor heterogeneity and multi-level regulation. In the context of BRCA, it is plausible that dysregulation of EP300 or HDAC activity contributes to the altered lactylation levels and downstream gene expression changes observed in our study. However, experimental validation, such as pharmacological inhibition of EP300 in breast cancer cell lines, is required to further elucidate the causal mechanisms.

In conclusion, our study provides a foundational framework for understanding lactylation in BRCA and supports its potential as a biomarker and therapeutic target. By linking metabolism with epigenetic and immune regulation, lactylation may open new avenues for precision oncology in BRCA.

## 5. Conclusions

In summary, this study demonstrates that lactylation is a critical regulator of breast cancer (BRCA) progression, influencing both tumor cell behavior and the immune microenvironment. We identified six lactylation-related hub genes—*ALDH1A1, HMGA1, DDX39A, KIF2C, HDGF, *and* S100A4*—that are strongly associated with immune cell infiltration and tumor proliferation. Single-cell transcriptomic analysis confirmed their heterogeneous expression across distinct BRCA cell populations, while in vitro functional assays revealed that elevated lactic acid levels enhance lactylation and promote the expression of oncogenic drivers, particularly S100A4. These findings indicate that lactylation is not merely a byproduct of altered metabolism, but functions as an active epigenetic modifier contributing to BRCA pathogenesis. Collectively, our study provides a novel mechanistic link between tumor metabolism and immune modulation, offering new avenues for therapeutic intervention in breast cancer.

## Figures and Tables

**Table 1:** List of PCR primers.

The PCR Primers		Sequence
Human-GAPDH	Forward	CAAGGTCATCCATGACAACTTTG
Human-GAPDH	Reverse	TCCACCACCCTGTTGCTGTAG
Human-RFC4	Forward	CAAGGATCGAGGAGTAGCT
Human-RFC4	Reverse	TTCCAGGTGGTCCGTAAA
Human-DDX39A	Forward	GACTCGGACACCTACCTGC
Human-DDX39A	Reverse	CTGCCACATTAACTTCAAACC
Human-HMGA1	Forward	AAGGACGGCACTGAGAAGC
Human-HMGA1	Reverse	CCTGGAGTTGTGGTGGTTTT
Human-KIF2C	Forward	TCAGAGCTTCGCATCACG
Human-KIF2C	Reverse	CATCTCCTCGCTGACCAT
Human-VIM	Forward	AATGGCTCGTCACCTTCG
Human-VIM	Reverse	AGTTTCGTTGATAACCTGTCC
Human-RACGAP1	Forward	TGGGCACGCCACAGAGTA
Human-RACGAP1	Reverse	GACAGCGGTCCCGACATT
Human-CRABP2	Forward	AGGGAGACACTTTCTACATCA
Human-CRABP2	Reverse	TCACCAGGCTCTTACAGG
Human-ALDH1A1	Forward	GGCAGCCATTTCTTCTCA
Human-ALDH1A1	Reverse	TGTCCAAGTCGGCATCAG
Human-SATB1	Forward	AGGTGTCTTCCGAAATCTA
Human-SATB1	Reverse	CAAGCCCTGAGTTCTGTT
Human-ECHDC1	Forward	GGCACAAGAATGGCTAAA
Human-ECHDC1	Reverse	CTGTAATGCTTCCTCCAA
Human-FABP5	Forward	TTTACAGATGGTGCATTG
Human-FABP5	Reverse	AGATCCGAGTACAGGTGA
Human-S100A4	Forward	GCCCTGGATGTGATGGTG
Human-S100A4	Reverse	CGTTGTCCCTGTTGCTGT

## Data Availability

The data presented in this study are openly available in TCGA-BRCA (https://portal.gdc.cancer.gov/projects/TCGA-BRCA, accessed on 3 April 2025).

## Supplementary Materials

The following supporting information can be downloaded at https://www.mdpi.com/article/10.3390/cimb48040416/s1.

## Abbreviations

The following abbreviations are used in this manuscript:

BRCA	Breast cancer
TFHC	T follicular helper cells
TME	Tumor microenvironment
ER	Estrogen receptors
PR	Progesterone receptors
HER2	Human epidermal receptor 2
CAFs	Cancer-associated fibroblasts
MDSCs	Myeloid-derived suppressor cells
MADD	MAP kinase-activating death domain protein
GO	Gene ontology
BP	Biological process
CC	Cell component
MF	Molecular function
DEGs	Differentially expressed genes
KEGG	Kyoto encyclopedia of genes and genomes
GSVA	Gene set variation analysis
PCA	Principal component analysis
DMEM	Dulbecco’s modified eagle medium
cDNA	Complementary DNA
RT-qPCR	Quantitative real-time PCR
