# Supplementary material for: Identification of Key Genes Regulated by Lactylation Modification and Associated with Tumor Immune Microenvironment in Breast Cancer
Source: Curr Issues Mol Biol. 2026 Apr 17;48(4):416. doi: 10.3390/cimb48040416 (PMC13114778; doi:10.3390/cimb48040416)
Supplement: Supplementary file 1 [file cimb-48-00416-s001.zip › cimb-4208959-supplementary/Supplementary Materials.pdf]

Figure S1: Differential gene expression and functional analysis of expressed genes between BRCA and normal samples. Figure S2: Enrichment and RT-qPCR validation of lactylation-related genes. Figure S3: Expression and prognostic significance of 6 lactylation-related genes in BRCA. Figure S4: Single-cell transcriptome analysis reveals gene expression patterns in different immune cell populations. Figure S5: Statistical test chart of Figure 6.
