# Supplementary figures and images for: Identification of Key Genes Regulated by Lactylation Modification and Associated with Tumor Immune Microenvironment in Breast Cancer
Source: Curr Issues Mol Biol. 2026 Apr 17;48(4):416. doi: 10.3390/cimb48040416 (PMC13114778; doi:10.3390/cimb48040416)

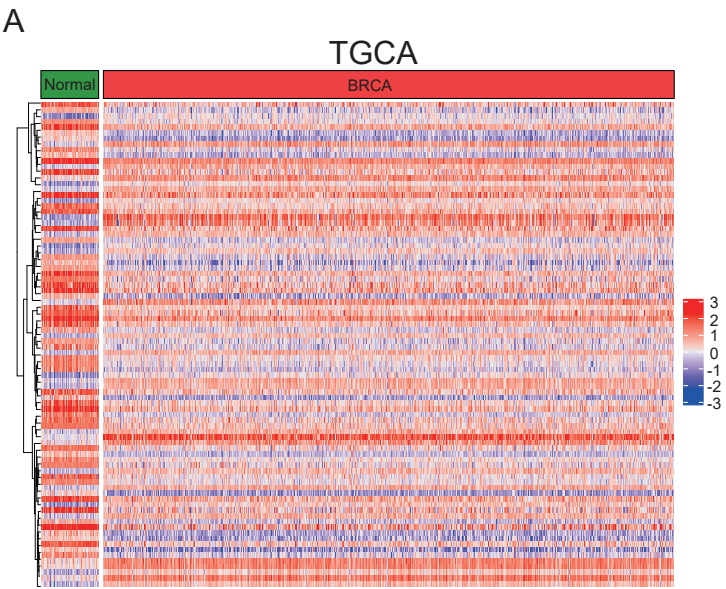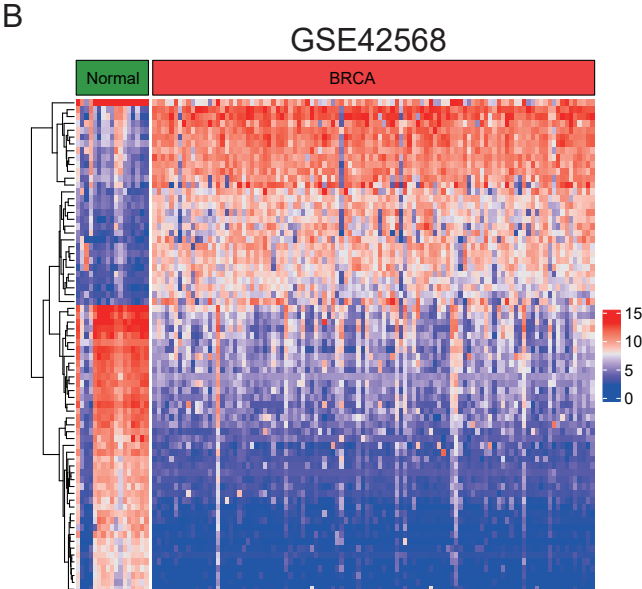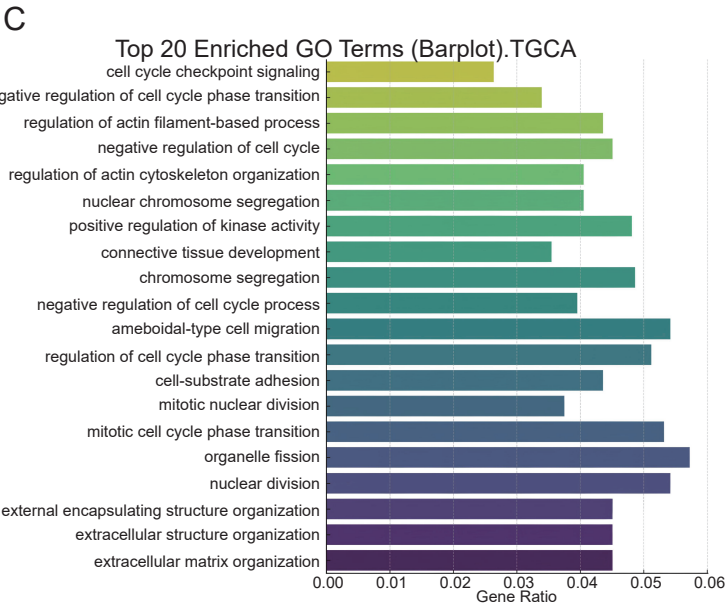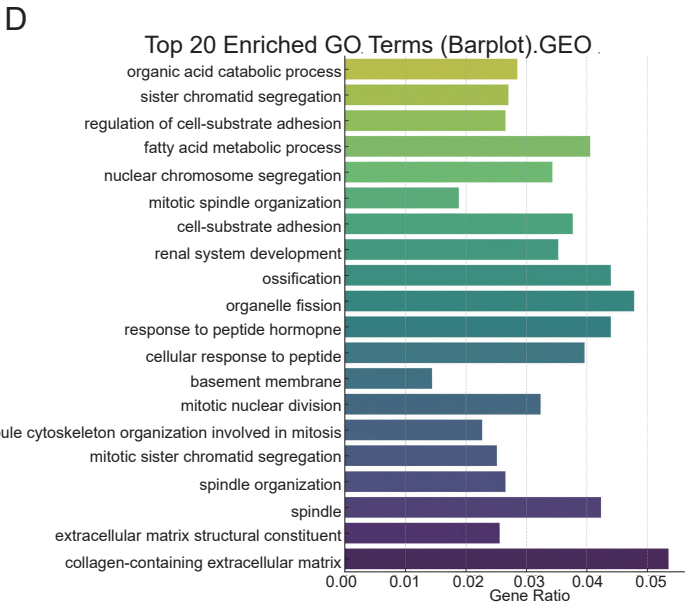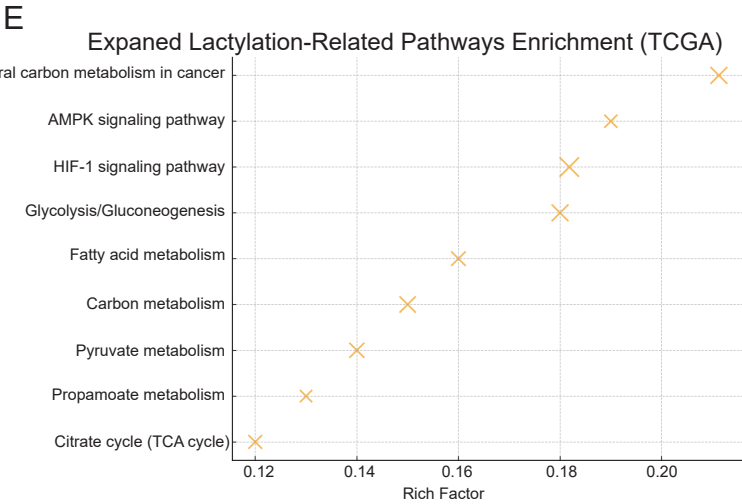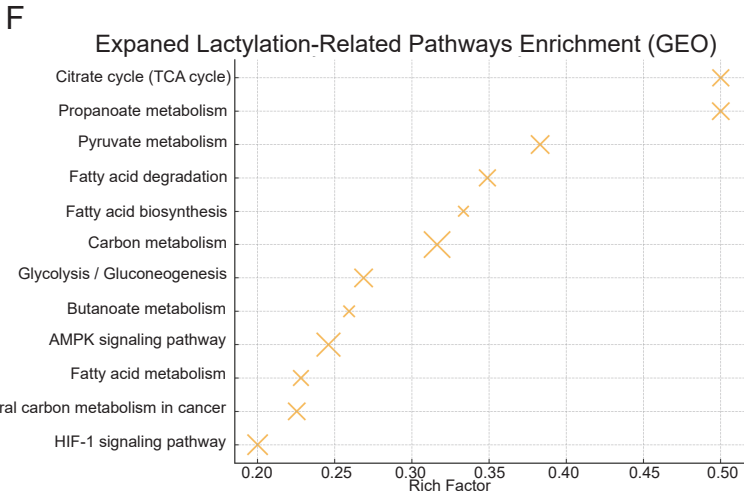

Supplement: Supplementary file 1 [file cimb-48-00416-s001.zip › cimb-4208959-supplementary/Figure S1.pdf]

A

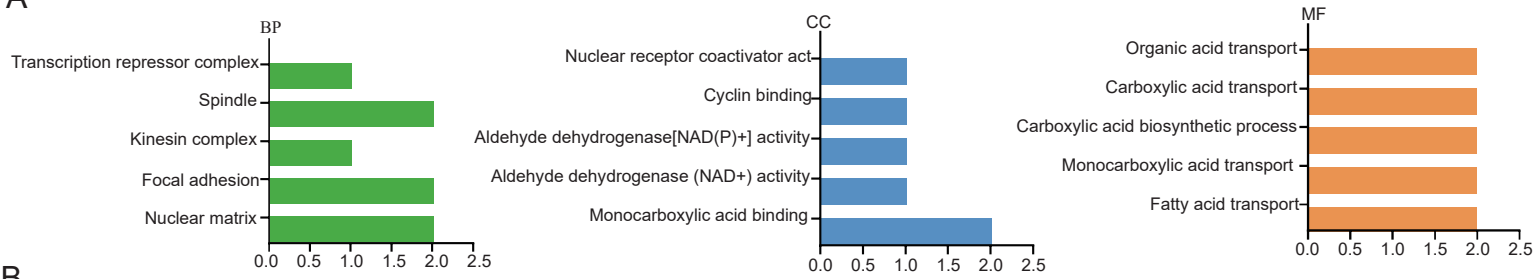

B

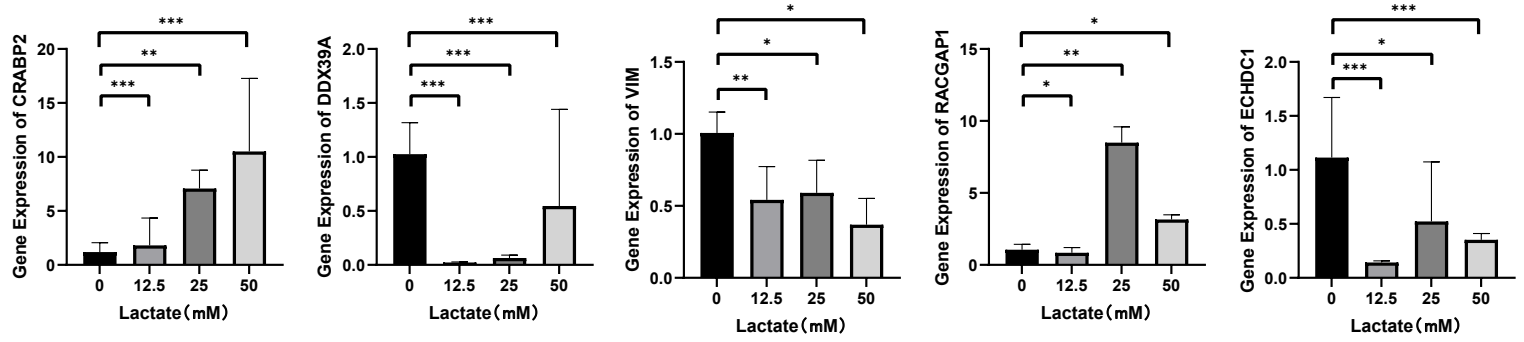

C

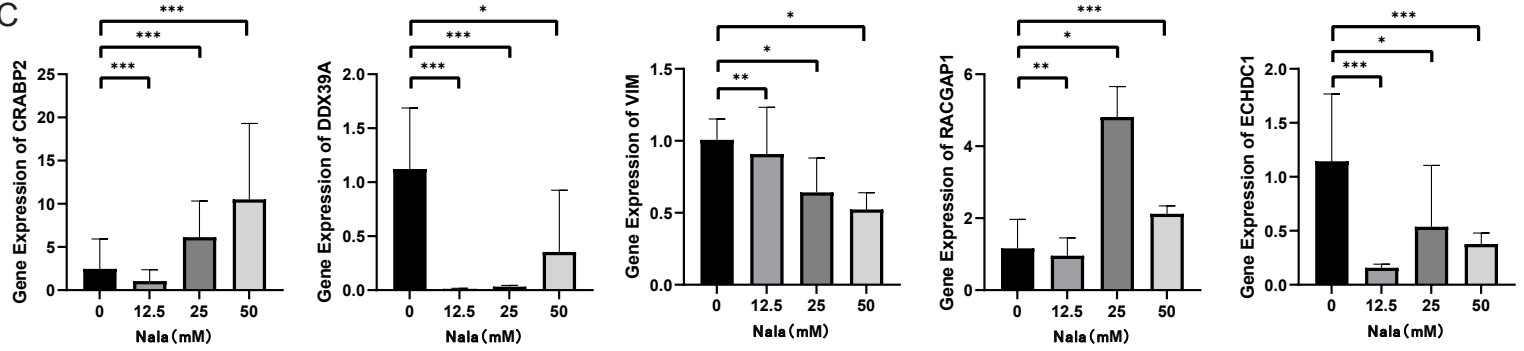

Supplement: Supplementary file 1 [file cimb-48-00416-s001.zip › cimb-4208959-supplementary/Figure S2.pdf]

A

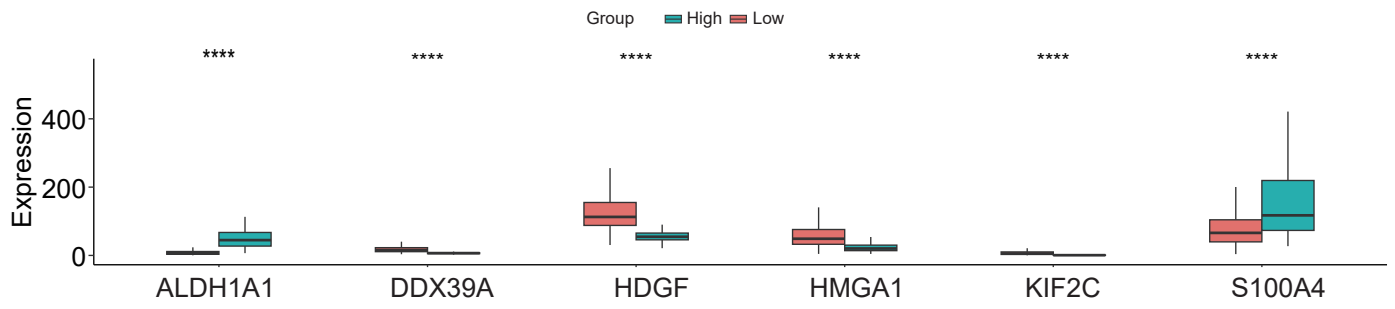

B

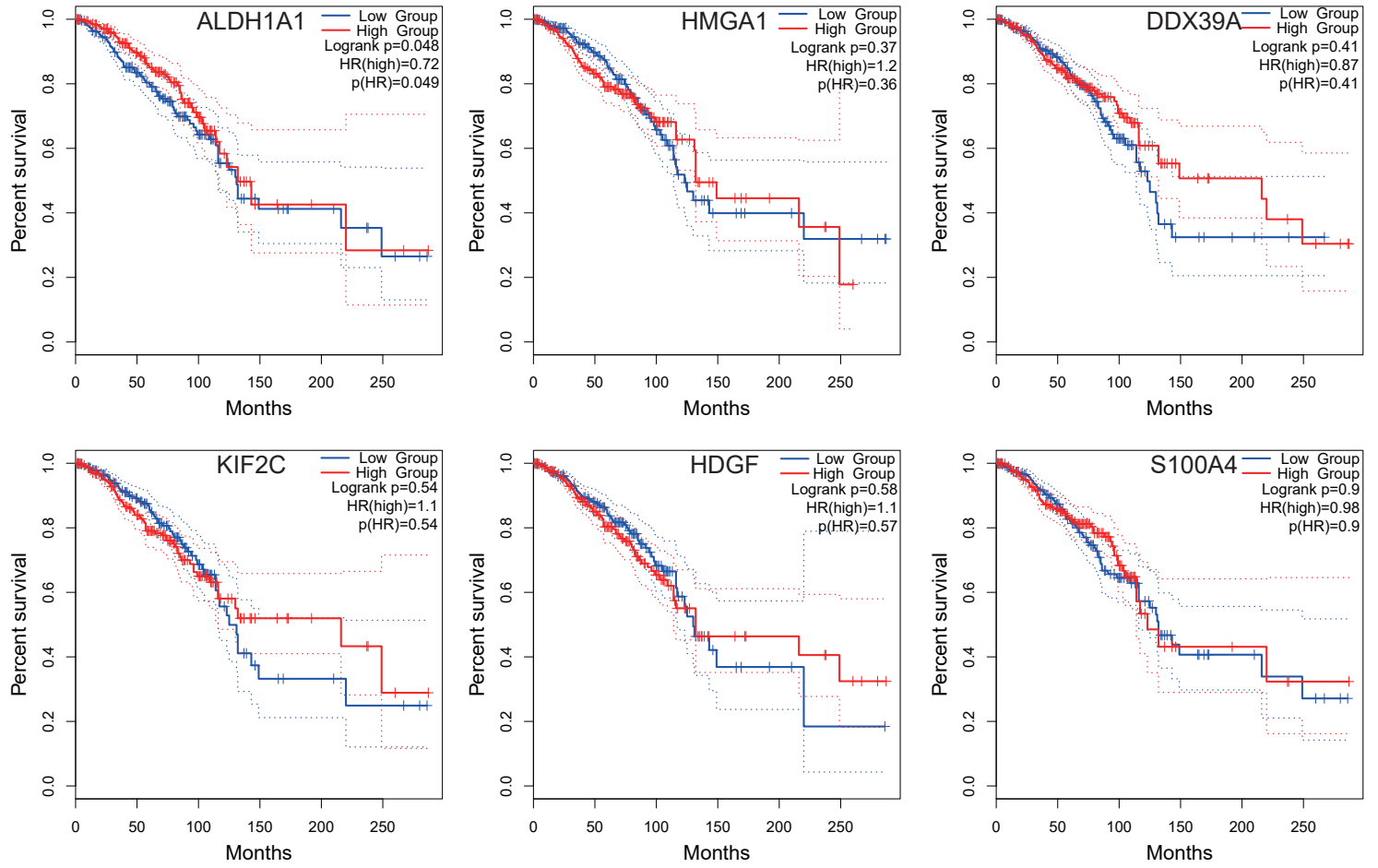

Supplement: Supplementary file 1 [file cimb-48-00416-s001.zip › cimb-4208959-supplementary/Figure S3.pdf]

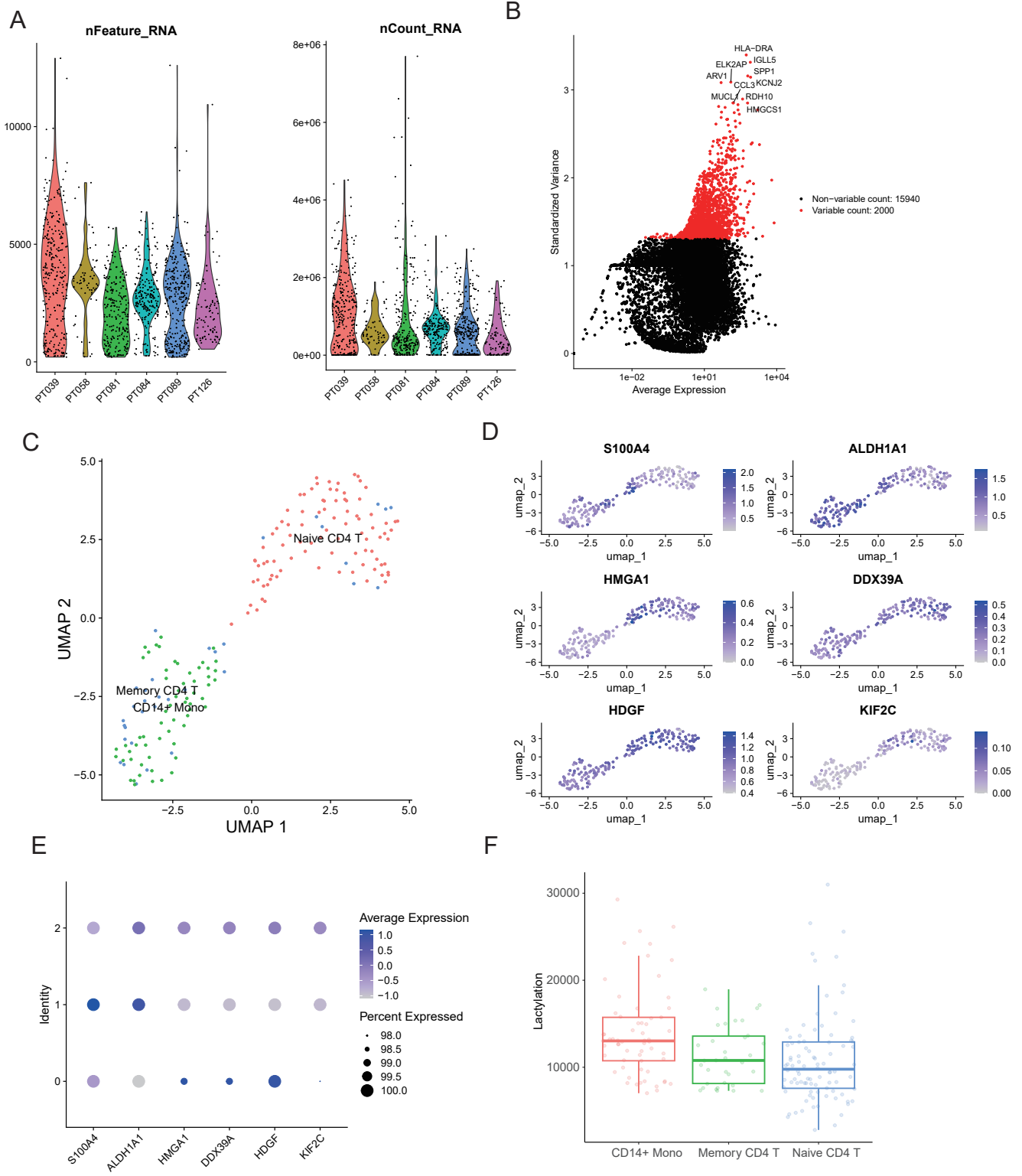

Supplement: Supplementary file 1 [file cimb-48-00416-s001.zip › cimb-4208959-supplementary/Figure S4.pdf]

A

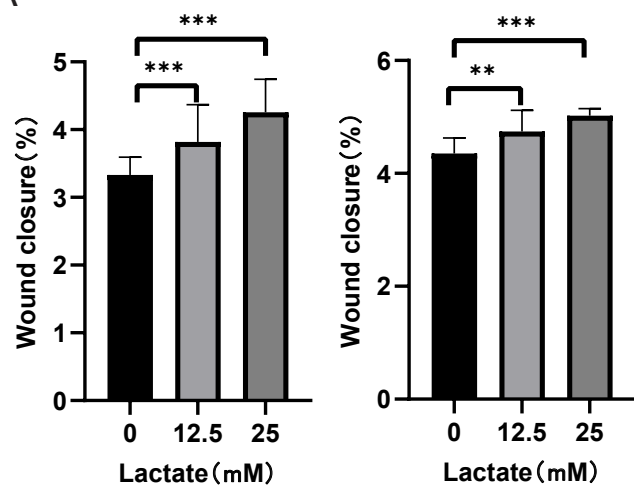

B

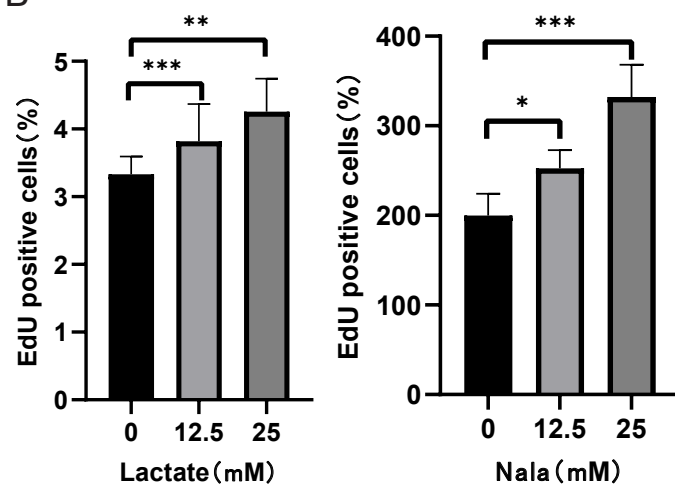

C

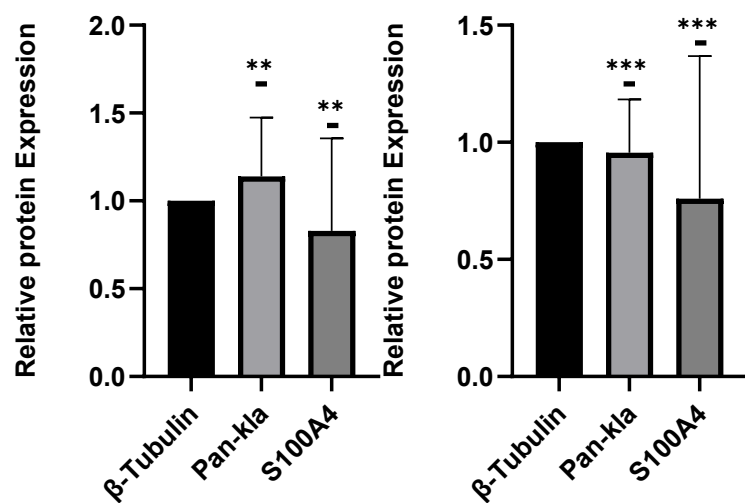

D

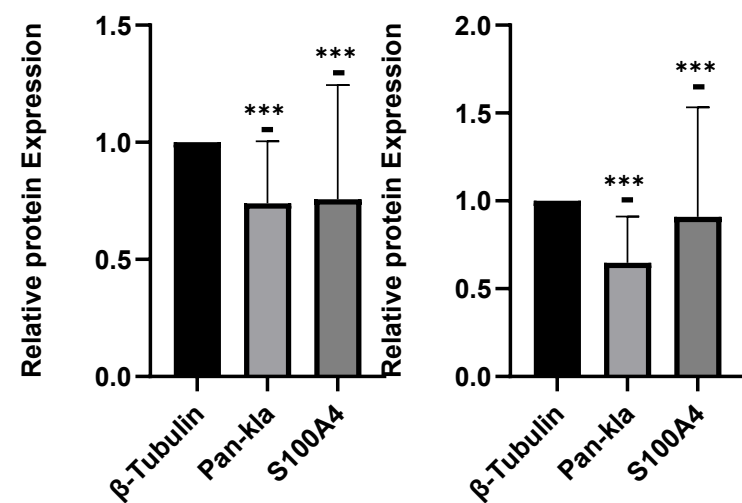

Supplement: Supplementary file 1 [file cimb-48-00416-s001.zip › cimb-4208959-supplementary/Figure S5.pdf]
